# Growth kinetics and characterization of human dental pulp stem cells: Comparison between third molar and first premolar teeth

**DOI:** 10.4317/jced.52824

**Published:** 2017-02-01

**Authors:** Davood Mehrabani, Parisa Mahdiyar, Kianoosh Torabi, Reza Robati, Shahrokh Zare, Mehdi Dianatpour, Amin Tamadon

**Affiliations:** 1 Assistant Professor of Stem Cell and Transgenic Technology Research Center, Shiraz University of Medical Sciences, Shiraz, Iran; 2 Assistant Professor of Department of Developmental Biology, Science and Research Branch, Islamic Azad University, Fars, Iran; 3 Assistant Professor of Department of Regenerative Medicine, University of Manitoba, Winnipeg, Manitoba, Canada; 4 Associate Professor of Department of Fixed Prosthodontics, Faculty of Dentistry, Shiraz University of Medical Sciences, Shiraz, Iran; 5 Assistant Professor of Department of Medical Genetics, School of Medicine, Shiraz University of Medical Sciences, Shiraz, Iran

## Abstract

**Background:**

Dental pulp stem cells (DPSCs) play an important role in tissue regeneration. This study compares the growth kinetics and characterization of third molar and first premolar human DPSCs.

**Material and Methods:**

Dental pulp tissues were isolated from human first premolar and third molar teeth and were digested by treating them with collagenase type I. Single-cell suspensions from each dental pulp were seeded in T25 culture flasks and the media were replaced every 3 days until 70% confluence. The cells were enumerated to determine the population doubling time (PDT). Cells were characterized using flow cytometry, RT-PCR and osteogenic medium for differentiation of DPSCs. Karyotyping assay was also performed till passage 7th.

**Results:**

The DPSCs had spindle-shaped morphology. There was an increase in PDT in third molar DPSCs when compared to first premolar teeth. Positive expression of CD44, CD73, and CD90 and negative expression of CD34 and CD45 were illustrated. A normal karyotype was visible for all seven passages. The Alizarin red staining was positive for osteogenic induction of DPSCs.

**Conclusions:**

When DPSCs are needed, third molar teeth can be a good and convenient candidate for cell transplantation, yielding high number of cells with mesenchymal characteristics. They can be a source for further investigations *in vitro* and work on tissue engineering protocols.

** Key words:**Stem cells, dental pulp, growth kinetics, characterization.

## Introduction

Isolation of mesenchymal stem cells (MSCs) has been reported from bone marrow (BM) (1), adipose tissue (2), endometrium (3), periodontal ligament (4), and dental pulp (5). MSCs are undifferentiated clonogenic cells capable of both self-renewal and multi-lineage differentiation (6) and their cell-based therapies are emerging as an alternative treatment choice for promotion of the functional recovery in patients suffering from several disorders that can be a major cause of death and permanent disability (7).

Multilineage properties of MSCs was shown to be dependent on the source and the donor which is responsible for their different behavior *in vivo* and their differentiation properties into mesodermal and ectodermal cellular lineages (8). Dental pulp stem cells (DPSCs) play an important role in tissue regeneration (9). Third molar tooth (10) and exfoliated deciduous teeth were reported as good sources of DPSCs (11). Presence of DPSCs in the pulp tissue of rat, mouse, canine, porcine, ovine, rabbit, chimpanzee, and rhesus has also been reported (12). There have been no systematic comparisons on DPSCs from different tooth sources. This study compared the growth kinetic and characterization of third molar and first premolar human DPSCs.

## Material and Methods

-Isolation of DPSCs

Third molar and first premolar teeth (Each: n=6) of 10-18 years old patients were obtained after extraction because of orthodontic reasons, under local anesthetic, with informed consent and institution ethical approval. Teeth roots were with viable pulp tissue. Dental pulp was pulled out and washed twice with sterile phosphate buffered saline (PBS; Gibco, USA) supplemented with antibiotics (100 U/ml penicillin and 100 µg/ml streptomycin) (Sigma, USA) and 2.5 µg/ml fungisone (Sigma, USA). Pulp tissue was minced into 1-2 mm fragments and were digested in a 3 mg/mL collagenase type I (Invitrogen, USA) solution for 30 min at 37ºC. They were transferred to T25 culture flasks containing Dulbecco’s Modified Eagle Medium (DMEM; Gibco, USA), 10% fetal bovine serum (FBS; Gibco, USA), 1% penicillin and streptomycin, 1% L-glutamine (Sigma, USA) and were cultured and incubated in a CO2 incubator at 37ºC with 5% CO2 and saturated humidity. The medium was replaced every 2 days and cells were subcultured at 80% confluence.

-Population doubling time

To enumerate the cells, DPSCs of third molar and first premolar (3×104, 6×104 and 11×104 cells/per well) at the seventh passage were seeded into 24-well culture plates. The cell number was assessed after 7 days by trypsinization (3 replicates for each time point). The cells were stained by trypan blue (Sigma, USA) and counted using a hemocytometer under a light microscope. The population duplication times (PDT), or the time required for a culture to double in number, was calculated with the following formula: PDT=T ln2/ln(Xe/Xb), T is the incubation time in hours, Xb is the cell number at the beginning of the incubation time and Xe is the cell number at the end of the incubation time.

-Cell viability

Trypan blue exclusion test (0.4% trypan blue in PBS) was performed for each passage to determine the number of viable and nonviable cells.

-Morphologic evaluation

DPSCs from both third molar and first premolar teeth, at each passage, were morphologically evaluated under inverted microscope (Olympus, Japan).

-Characterization by flow cytometry

After harvesting, DPSCs (4th to 7th passage) were washed in cold PBS supplemented with 0.5% BSA (Sigma-Aldrich, Saint Louis, MO, USA). Aliquots of 5×105 cells were labeled (30 min in the dark at 4oC) with monoclonal antibodies specific for human markers associated with mesenchymal and hematopoietic lineages. Namely, mouse antihuman antibodies against the following antigens were used: FITC-labeled anti-CD34 (1:20; DAKO, Carpinteria, CA, USA), and anti-CD44 and anti-CD90 (1:20; DAKO). To determine the level of nonspecific binding, fluorochrome conjugated isotype control antibodies (BD Biosciences, Heidelberg, Germany) were used. Flow cytometry was performed using a CyFlow CL (Partec, Münster, Germany).

-Characterization by RT-PCR

To determine the expression of mesenchymal stem cell markers, RT-PCR was performed for DPSCs of all teeth. First, total RNA was extracted using the column RNA isolation kit (Denazist-Asia, Iran) in accordance with the manufacturer’s instructions. Total RNA concentration was evaluated by spectrophotometer. Then, complementary DNA (cDNA) was provided from RNA samples using AccuPower Cycle Script RT PreMix Kit (Bioneer, Korea) according to the manufacturer’s instructions.

Briefly, 15 µL of total RNA was used for each reaction and the volume reached up to 20 µL with the DEPC water. Twelve thermal cycles was performed as follows: 30 sec at 20ºC for primer annealing, 4 min at 42ºC for cDNA synthesis, 30 sec at 55˚C for melting secondary structure and cDNA synthesis and 5 minutes at 95ºC for inactivation. In the third step, 1 µL of template (cDNA) was mixed with other reagents consisting of PCR buffer, MgCl2, H2O, dNTPs, Taq DNA polymerase, and forward and reverse primers (H45 and H73).

Then, the microtubules containing 20 µL of the above mixture were put in thermocycler (Eppendorf Mastercycler Gradient, Eppendorf, Hamburg, Germany). Thirty amplification cycles were run, consisting of 30 sec denaturation at 95ºC, 30 sec annealing at 64ºC and 30 sec extension at 72ºC with the 2 min at 95ºC for primary denaturation and 5 min at 72ºC for final extension. PCR products were evaluated for the presence of considered bands by gel electrophoresis with the aid of DNA safe stain in 1.5% agarose gel medium. Produced bands were visualized under UV radiation by Gel documentation system (UVtec, Cambridge, UK).

-Karyotype analysis

Karyotyped DPSCs (subcultured at a 1:3 dilution, both early passages and after reaching Hayflick’s limit) were subjected to a 4-hour demecolcemide (Sigma, USA), incubated for 24 hours and then addition of trypsin-EDTA (Sigma, USA) and hypotonic KCl (Sigma, USA) and finally fixation in acid/alcohol solution. The number of chromosome was determined under a microscope with an oil immersion objective. Images were captured using digital camera and light microscopy to analyze the metaphase.

-Osteogenic induction 

Approximately 1×104 cells of DPSCs were transferred into two 35 mm culture dishes (Corning, Germany) including the control media composed of DMEM-F12, (Bio West, France) supplemented with 10% FBS (FBS, BioIdea, Iran), 1% penicillin/streptomycin and 1% L-glutamine (BioIdea, Iran) and the osteogenic media consisted of DMEM-F12, 10% FBS, 1% penicillin-streptomycin, L-glutamine, 50 μg/ml L-ascorbic acid-2-phosphate (Sigma10-7 M dexamethasone (Sigma, USA), and 10 mM β-glycerophosphate (Sigma, USA). The medium was replaced every 3 days. On day 21, cultures were fixed with 70% ethanol for 15 min and stained for mineralization with 2% alizarin red S. The cell layers were then evaluated with light microscope.

## Results

-Growth curve of DPSCs

**Figure 1:**
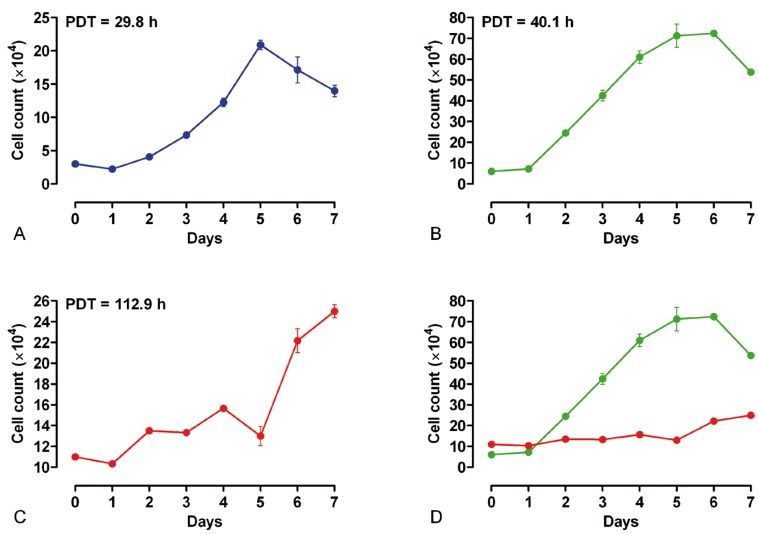
Growth curves and PDT of DPSCs isolated from human. A) third molar tooth (with initial density of 3×104 cells per well); B) first premolar tooth (with initial density of 6×104 cells per well); C) first premolar tooth (with initial density of 11×104 cells per well) and D) a comparison between growth curves of both cultures initiated with two different numbers of cells, 6×104 (green line) and 11×104 (red line) cells per well.

Growth curves and population doubling times (PDT) of the seventh passage of DPSCs when seeding different densities of cells per well in 24 well culture plates were shown in figures 1A-D. The growth curve of DPSCs isolated from human third molar teeth while the initial density of seeded cells per well were 3×104 cells revealed that the PDT was 29.8 hours (Fig. 1A). The growth curve of DPSCs isolated from human first premolar teeth at 6×104 cells per well initially seeded denoted to the PDT was 40.1 hours (Fig. 1B) showing that DPSCs isolated from human third molar teeth proliferated much more faster.

When for first premolar teeth, the initial density of seeded cells per well was 11×104 cells, the PDT increased to 112.9 hours (Fig. 1C). The comparison of growth curve of first premolar teeth with different initial density of seeded cells of 6×104 (green line) and 11×104 (red line) cells per well demonstrated that the cell proliferation rate was significantly more in DPSCs isolated from human third molar teeth (Fig. 1D).

-Morphology of DPSCs

**Figure 2:**
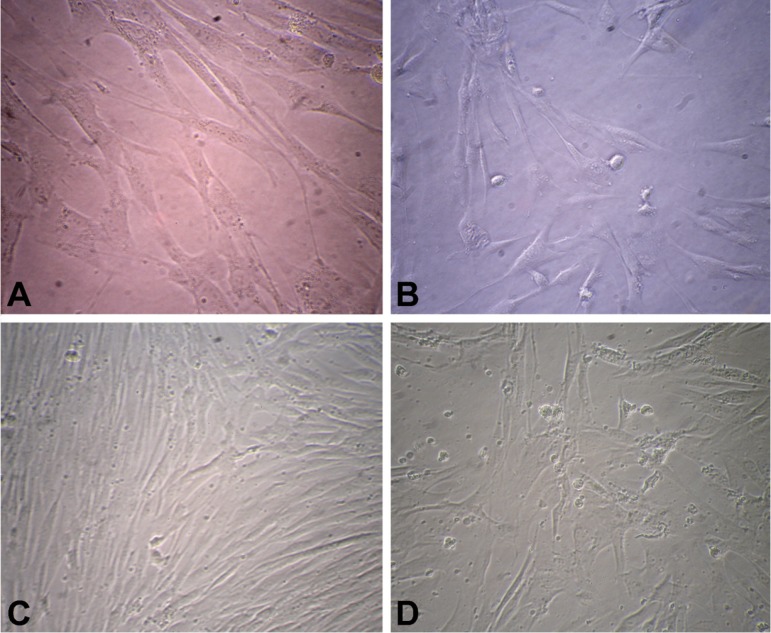
Morphology of human DPSCs isolated from different sources. A) primary culture of first premolar teeth (×40); B) DPSCs isolated from first premolar in passage 7 (×40); C) DPSCs isolated from primary culture of third molar teeth (×20); and D) DPSCs isolated from third molar in passage 7 (×20).

Ten days after expansion of DPSCs, both third molar and first premolar teeth showed a fibroblast like, elongated, spindle shaped morphology and adherent property under a convert microscope at all passages (Fig. 2).

-Characterization by flow cytometry

**Figure 3:**
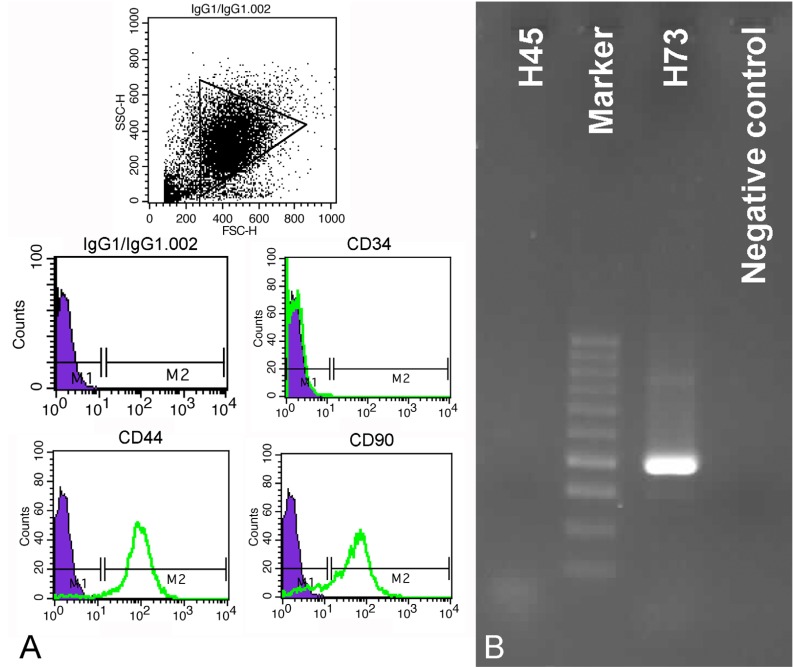
Characterization of DPSCs by flow cytometry and RT-PCR. A) Cells were uniformly negative for CD34, and positive for CD44 and CD90. B) Positive expression of CD73 compared with negative expression of CD45 using RT-PCR technique.

DPSCs isolated from both third molar and first premolar teeth were positive for expression of CD44 and CD90 and negative for CD34 expression (Fig. 3A).

-Characterization by RT-PCR

Using RT-PCR, it was shown that all cells till passage 7 expressed CD73. CD45 was not expressed in any of DPSCs (Fig. 3B).

-Karyotype analysis

**Figure 4:**
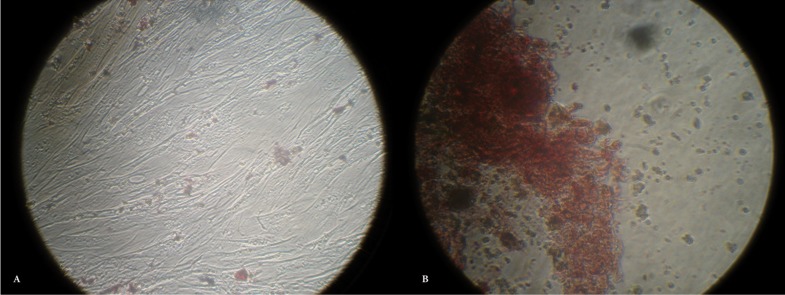
The chromosome number of DPSCs isolated from both third molar and first premolar teeth (2n=46, 44 autosomal and 2 sex chromosomes, Figure 4A and 4B, respectively).

The chromosome number of DPSCs isolated from both third molar and first premolar teeth was 2n=46, containing 44 autosomal and 2 sex chromosomes in all passages (Fig. 4A, 4B, respectively).

-Osteogenic induction 

**Figure 5:**
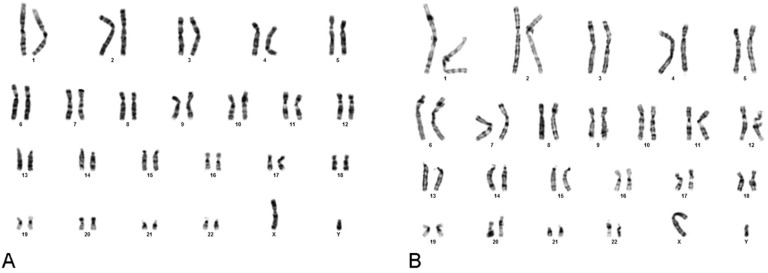
Analysis of alizarin red S staining of DPSCs isolated from both third molar and first premolar teeth at passage 7 (osteogenic medium: Figure 5A and 5B).

Analysis of alizarin red S stained areas demonstrated the osteogenic potential of DPSCs isolated from both third molar and first premolar teeth at passage 7 (osteogenic medium: Fig. 5A,B).

## Discussion

Our study showed that DPSCs isolated from both third molar and first premolar teeth were mesenchymal stem cells with spindle shape morphology, multipotency, and expression of mesenchymal and lack of hematopoietic stem cell markers (13). Considering growth kinetic of DPSCs, the difference between the growth curves of DPSCs from third molar and deciduous teeth was previously investigated (14). The properties of permanent teeth in regenerative medicine was previously investigated and the advantages of deciduous teeth as a new source of stem cells were demonstrated (15).

In contrast, Eslaminejad *et al.* demonstrated that DPSCs were isolated from third molar had better growth rate versus DPSCs isolated from deciduous teeth which is similar to our findings (16). Comparison of growth curves indicated that the proper density of initial cell number for cell culturing could be considered as a significant parameter for cell culturing which was also noticed in our study (17). An increase in cell density during seeding was shown to display a negative role in cell proliferation (18). We also illustrated that initial number of 6×104 cells per well was an adequate number for cell culture and expansion. There has been no comparisons in literature to evaluate the growth kinetic of DPSCs from third molar and first premolar teeth. Our present study showed that DPSCs of third molar teeth had a lower PDT and proliferated faster than DPSCs from first premolar teeth.

Regarding characterization of DPSCs by flow cytometry, we demonstrated that DPSCs isolated from both third molar and first premolar teeth were positive for expression of CD44 and CD90 and negative for CD34 confirming mesenchymal property of DPSCs (13,18,19). In our study when using RT-PCR, it was shown that DPSCs expressed CD73 and lacked expression of CD45 which confirm mesenchymal properties of DPSCs as reported before (20).

The differentiation potential of harvested DPSCs has an important role in tissue engineering. We showed that DPSCs from both third molar and first premolar teeth were positive for osteogenic differentiation and successfully differentiated into osteoblasts (11). Genetic stability is considered an important factor in cell culture in different passages and to obtain a normal cell line (17). We noticed a normal karyotyping for DPSCs from both third molar and first premolar teeth up to 7th passage. The successful clinical use of DPSCs in different trials for bone repair showed that DPSCs can be an appropriate cell source for transplantation (16) with immune-modulating properties in cell therapies (7).

DPSCs are derived from various dental tissues such as human exfoliated deciduous teeth, apical papilla, periodontal ligament and dental follicle tissue (21). In our study, the harvested cells were able to expand up to passage 7 and were positive for mesenchymal cell markers and with multi-lineage differentiation properties as described before to have a MSCs phenotype and differentiation properties into chondrocytes, osteoblasts, cardiomyocytes, neuron, liver cells and β cells of islet of pancreas (21).

## Conclusions

We have compared the PDT of DPSCs of third molar teeth with first premolar teeth and found that DPSCs from third molar teeth proliferated much faster. So we can conclude that if DPSCs are searched for cell therapy purposes in dental related problems, DPSCs of third molar teeth can be a good candidate and convenient source for cell transplantation, tissue engineering and regenerative medicine based on their short PDT.
